# The Translational Value of Preclinical Research: Lessons from More Than Forty Years of Retinal Research

**DOI:** 10.3390/biomedicines14102405

**Published:** 2026-10-21

**Authors:** Paola Bagnoli

**Affiliations:** Department of Biology, University of Pisa, 56127 Pisa, Italy; paola.bagnoli@unipi.it

**Keywords:** retinal research, translational medicine, preclinical models, somatostatin, uPA/uPAR, β-adrenoceptors, drug repurposing, drug delivery

## Abstract

The present paper is based on a long personal experience of research on the retina. Over almost forty years, questions that were originally pursued as curiosity-driven physiology progressively acquired unexpected translational implications. The retina provides a particularly instructive setting in which to examine translational potential because its accessible and highly organized neural and vascular architecture has allowed mechanisms identified in experimental models to be tested as potential therapeutic targets. Three research trajectories from our laboratory are discussed as examples of different translational outcomes. Studies of the somatostatinergic system illustrate how a strong mechanistic rationale and convincing preclinical efficacy may not necessarily translate into comparable clinical benefit. Investigations of the uPA/uPAR system and its pharmacological inhibition show how a candidate that does not reach clinical application may nevertheless provide durable insight into disease mechanisms and identify biologically relevant pathways. Conversely, studies of β-adrenoceptor signaling and propranolol in retinopathy of prematurity illustrate how drug repurposing can shorten the distance between experimental evidence and clinical investigation, while still leaving important questions about dose, safety, and delivery. These experiences also highlight model selection, disease stage, pharmacokinetics, tissue exposure, reproducibility and patient heterogeneity as critical determinants of translational success. Taken together, my experience of research on the retina suggests that translation should be viewed not as a one-way progression from bench to bedside, but as an iterative process in which experimental findings, clinical outcomes and even unsuccessful therapeutic attempts continuously reshape biological questions.

## 1. Introduction

The path from basic biological discovery to clinical application is rarely linear, and the scientific value of preclinical research cannot be judged solely by whether an experimental compound eventually becomes an approved type of therapy. Over the years, broad questions in retinal research have gradually been complemented by increasingly specific efforts to identify the molecular and cellular players that regulate distinct functional processes. A representative example is optic nerve (ON) injury: more than a century after Santiago Ramón y Cajal described the failure of retinal ganglion cell (RGC) axons to regenerate after ON damage, the mechanisms that might permit meaningful axonal regeneration are still being defined. After severe ON damage, as occurs in optic neuropathies, promoting RGC axon regeneration is vital for restoring vision. Although many genes and key molecules have been identified for their ability to support RGC survival, current therapies for glaucoma, a neurodegenerative disorder marked by RGC loss and axonal degeneration, are still focused on lowering intraocular pressure to prevent further ON damage, using eye drops, laser procedures, or surgery. The translation of these discoveries into restorative treatment is still at an early stage [1].

It is only relatively recently that researchers have begun to attribute explicit translational value to their preclinical findings, but the question remains whether preclinical research necessarily has immediate translational value. Indeed, most preclinical candidates never become approved therapies, but this does not necessarily mean that the underlying research has failed [2,3].

This Perspective is intentionally selective rather than comprehensive. The three research programs discussed below were chosen because they arose directly from the author’s laboratory experience and represent three distinct translational trajectories: a biologically compelling target that reached clinical testing without entering routine retinal care, a novel candidate that remained in preclinical development, and a repurposed drug that progressed to early clinical investigation. The literature was selected to document the original mechanistic observations, the principal preclinical efficacy studies, and the clinical studies that determined the translational status of each program. The evidence is therefore interpreted according to level: mechanistic studies establish biological plausibility; animal studies provide proof-of-concept efficacy; exploratory or pilot clinical studies provide preliminary human signals; randomized controlled trials provide stronger evidence of efficacy and safety; and an established clinical benefit requires reproducible clinical evidence that is sufficient to support routine use. Negative, contradictory, and inconclusive findings were considered alongside positive results.

## 2. Why the Retina Became an Ideal Laboratory for Studying Translation

Translation in biomedical research is particularly attractive for the retina, which offers a useful model in which to examine the passage from discovery biology to therapeutic development. The retina has repeatedly served as a bridge between neuroscience, vascular biology and clinical ophthalmology because of its accessibility, defined cellular organization, measurable function, vascular architecture, and the possibility of testing local as well as systemic interventions. As an extension of the brain, the retina is an ideal model for translational research. Indeed, in preclinical studies, retinal manipulations provide consistent support for deciphering the mechanisms underlying ocular diseases and eventually identifying potential solutions.

The retina is experimentally accessible and occupies a defined position between the vitreous body and the retinal pigment epithelium. ON injuries represent classic models for investigating neurodegeneration and repair in the central nervous system. Retinal manipulations include, among others, intravitreal injections of drugs, viral vectors carrying genetic material, or other agents that are considered potentially useful for retinal treatment. The retina possesses a highly organized blood supply that optimizes oxygenation, allowing for high metabolic demand by its cells. At the functional level, light passes through the inner retinal layers to reach the photoreceptors, which convert it into electrochemical signals. A well-controlled input such as a light stimulus allows investigators to examine precisely how the retina converts incoming light into neural signals that reach the brain through the ON. Its comparatively compact circuitry retains many of the neuronal, glial, vascular, and signaling features of the central nervous system, while remaining sufficiently organized to permit precise experimental analysis.

## 3. Anti-VEGF Therapy as a Benchmark: Successful Translation, but Not a Complete Solution

Over the years, while studying the basic principles of retinal physiology, we uncovered distinct systems governing retinal angiogenesis, a critical process that satisfies the high oxygen and nutrient demands of the retina. Pathological neovascularization occurs in response to diverse disease-specific stressors, including hyperglycemia in proliferative diabetic retinopathy (PDR) and oxidative and degenerative changes in age-related macular degeneration (AMD), conditions that increasingly affect older populations who are often burdened by multiple chronic and multisystemic diseases. Proliferation of fragile, leaky vessels lacking normal tight junctions can cause fluid leakage, hemorrhage, tractional retinal detachment, and ultimately vision loss. Because alterations in retinal vascularization are closely coupled to retinal cell degeneration in major retinal diseases, translating knowledge of the systems that regulate retinal angiogenesis into retinal care has become increasingly relevant. Here, we address selected systems that, in our experience, contribute to pathological retinal or choroidal neovascularization in major sight-threatening diseases. While investigating potential strategies against pathological angiogenesis, we also encountered their possible neuroprotective actions [4].

Neovascularization frequently involves increased vascular endothelial growth factor (VEGF) signaling in response to hypoxia, ischemia, inflammation, or tissue stress. In diabetes, chronic hyperglycemia damages retinal microvessels and promotes ischemia; in older adults, age-related degenerative and vascular changes in the outer retina, retinal pigment epithelium, and choroid contribute to the development of nAMD. Conversely, in preterm infants, retinopathy of prematurity (ROP) has emerged as a leading cause of childhood blindness as advances in neonatal care have improved survival rates of premature babies. In the immature retina, the sudden transition from the relatively hypoxic in utero environment to the oxygen-rich extrauterine condition causes premature cessation of normal vessel growth, followed by persistent ischemia and progressive hypoxia in the peripheral retina. This triggers the upregulation of proangiogenic factors, including VEGF, and may result in abnormal, sight-threatening neovascularization. Proliferative and ischemic retinopathies therefore represent an increasing clinical burden. Their growing prevalence is driven in part by systemic diseases such as diabetes, in which both PDR and diabetic macular edema (DME) represent major vision-threatening complications. In addition, AMD is a leading cause of irreversible visual impairment in older adults in high-income countries, with age, smoking, genetic susceptibility, and cardiovascular risk factors contributing to disease risk. Demographic aging and lifestyle changes, including sedentary behavior and high-calorie diets, contribute to the increasing prevalence of multisystemic diseases associated with retinal vascular complications. Ischemic retinopathies are also increasing in low- and middle-income countries, where adoption of Westernized lifestyles and improved survival of premature infants have increased the disease burden, while access to specialized ophthalmic care may remain limited [4].

Anti-VEGF drugs have revolutionized the treatment of previously incurable diseases. Anti-VEGF drugs act by preventing VEGF from interacting with its receptors on endothelial cells, either by binding VEGF or by acting as decoy receptors with a high affinity for VEGF. Because the underlying tissue may remain hypoxic and continue to produce VEGF after treatment, repeated anti-VEGF injections are often necessary to suppress new vessel growth and leakage. Inflammation is an additional driver of proliferative retinopathies. In AMD, complement activation, macrophage recruitment, and cytokine signaling contribute to drusen formation, geographic atrophy, and choroidal neovascularization (CNV); choriocapillaris impairment and reduced choroidal perfusion may contribute to local hypoxia and VEGF induction within a broader multifactorial pathogenic process. In PDR, hyperglycemic metabolic stress leads to microglial activation, leukocyte accumulation, and elevated levels of inflammatory mediators, which in turn promote vascular leakage and pathological neovascularization. These mechanisms are not fully controlled by VEGF inhibition alone, and considerable work is therefore directed toward longer-acting anti-VEGF treatments and complementary anti-inflammatory or anti-fibrotic strategies [5,6].

Among currently available anti-VEGF treatments, aflibercept is a recombinant fusion protein that acts as a soluble decoy receptor and binds VEGF-A, VEGF-B and placental growth factor. Faricimab is a bispecific antibody targeting both VEGF-A and angiopoietin-2 (Ang-2), thereby combining anti-angiogenic activity with modulation of vascular instability. These molecular designs illustrate an important translational principle: target breadth and molecular architecture can influence both efficacy and durability. Clinical trials have shown that extended dosing intervals are feasible in many patients with neovascular AMD or DME [6]. Despite encouraging preclinical results and the increasing number of molecular targets explored by basic research, the transition of novel drugs into clinical trials remains highly challenging. Drug repurposing, such as the ophthalmic use of bevacizumab originally developed for cancer, provides an alternative route to clinical application [5].

Although anti-VEGF therapy has transformed the management of neovascular retinal disease, important limitations remain, including the treatment burden associated with repeated intravitreal injections, incomplete or variable responses, and the contribution of inflammatory and fibrotic pathways that are not directly controlled by VEGF inhibition [6].

Limitations of anti-VEGF treatment have stimulated intensive exploratory research aimed at developing novel approaches that retain therapeutic efficacy while reducing treatment burden and other limitations. In this context, although basic research continues to generate numerous candidate molecular targets for antiproliferative therapies, VEGF remains a leading target, while alternative strategies have shown mixed success. Among recent advances, faricimab neutralizes both VEGF-A and Ang-2, illustrating how dual-pathway inhibition may improve disease control beyond VEGF blockade alone [6].

Although established anti-VEGF agents dominate the treatment of neovascular AMD, additional therapies are still needed, and the cost and complexity of clinical development can slow the progression of promising candidates. Looking to the future, one promising strategy is sustained intraocular anti-VEGF activity through gene therapy, using viral vectors to deliver genetic material that enables retinal cells to produce anti-VEGF proteins locally. However, potential challenges might include long-term safety, immune responses to the vector, and managing surgical risks. Although gene therapy for neovascular retinal disease is advancing clinically, many compounds with anti-angiogenic or anti-inflammatory activity continue to show promise only at the preclinical level and do not progress to advanced clinical trials [5].

### Pegaptanib (Macugen): From SELEX to the First Anti-VEGF Aptamer Therapy

The development of pegaptanib provides an instructive historical example of how a basic molecular-selection technology can be translated into an ocular therapy. Systematic Evolution of Ligands by Exponential Enrichment (SELEX) was introduced in 1990 as an iterative procedure in which very large pools of nucleic-acid sequences undergo repeated rounds of target binding, partitioning and amplification, progressively enriching high-affinity ligands [7]. The application of this strategy to VEGF generated RNA aptamers with high affinity for VEGF165. Subsequent chemical optimization, including 2′-fluoropyrimidine and 2′-O-methyl substitutions to increase nuclease resistance and PEGylation to prolong intraocular persistence, produced the 28-nucleotide aptamer pegaptanib, which selectively binds the heparin-binding VEGF165 isoform rather than broadly neutralizing all VEGF-A isoforms [8].

**Figure 1:**
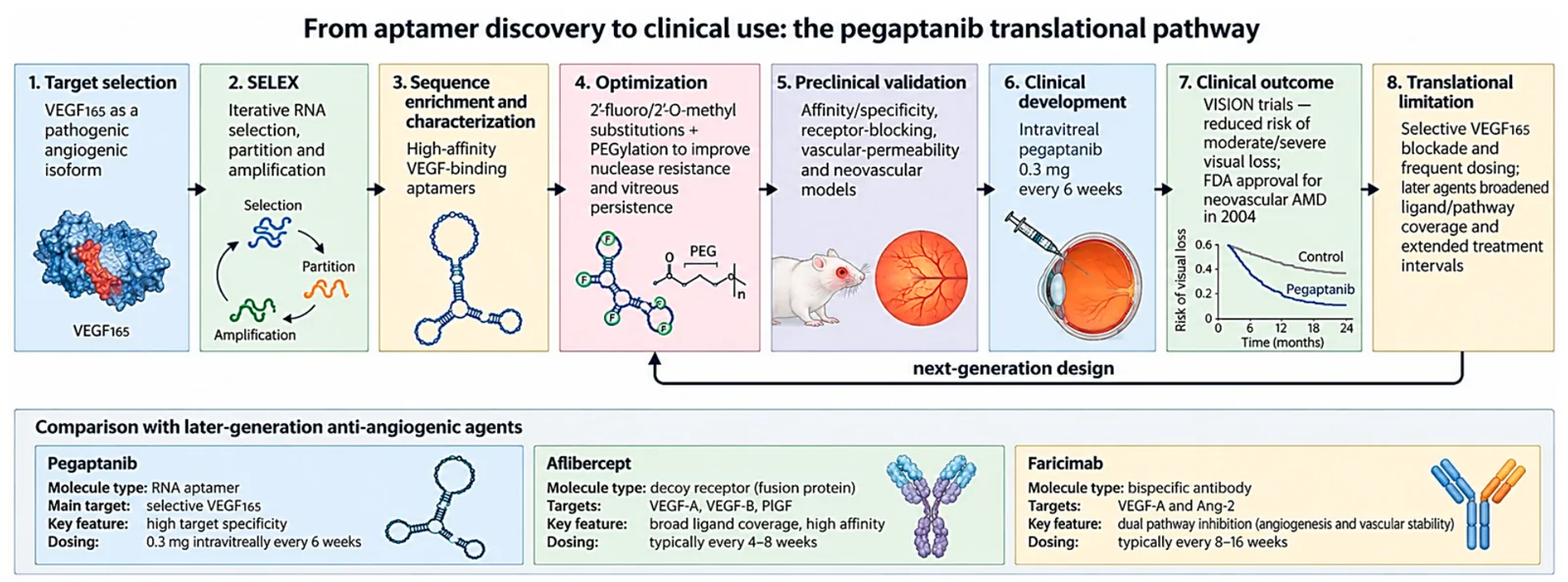
From aptamer discovery to clinical use: the pegaptanib translational pathway. SELEX enabled the enrichment of high-affinity RNA ligands against VEGF165, followed by sequence optimization and chemical modifications that improved nuclease resistance and intraocular persistence. Preclinical validation supported intravitreal clinical development, and the VISION trials established the efficacy of pegaptanib in neovascular AMD. The pathway also illustrates translational limitations related to selective VEGF165 coverage and repeated dosing, which helped motivate later anti-angiogenic designs with broader ligand or pathway coverage [6,7,8,9]. The figure is schematic; the clinical-outcome panel is illustrative and does not reproduce patient-level trial data.

The VISION program then translated this molecular concept into two concurrent randomized, double-masked, multicenter trials enrolling 1186 patients with neovascular AMD. Intravitreal pegaptanib at 0.3, 1.0 or 3.0 mg was administered every 6 weeks; the primary endpoint was the proportion of patients losing fewer than 15 letters of visual acuity at 54 weeks. With the approved 0.3 mg dose, 70% of patients lost fewer than 15 letters compared with 55% of sham-treated controls, while severe visual loss (≥30 letters) occurred in 10% versus 22%, respectively [9]. This program led to regulatory approval in 2004 and established that an RNA aptamer could become a clinically useful ophthalmic drug. At the same time, pegaptanib illustrates the limitations of highly selective target coverage and relatively frequent intravitreal dosing. Later agents were developed to broaden ligand or pathway coverage—aflibercept through decoy-receptor binding of VEGF-A, VEGF-B and placental growth factor, and faricimab through dual VEGF-A/Ang-2 inhibition—and contemporary regimens can permit longer treatment intervals in selected patients [5,6]. Cross-trial efficacy comparisons should nevertheless be interpreted cautiously because patient populations and retreatment algorithms differ. Figure 1 summarizes this discovery-to-clinic pathway and the translational design choices that shaped subsequent anti-angiogenic therapy.

## 4. Three Research Trajectories from Basic Biology Toward the Clinic

We approached preclinical studies in animal models of retinal disease by manipulating biological pathways in vivo in order to understand how they function. In those days, we were far from considering how the results of our research might be translated clinically. Indeed, exploring fundamental scientific questions without practical, real-world applications, such as medical treatments or technologies, was considered valuable in itself regardless of immediate outcome. The concept and organized framework of translational research gained prominence in the 1990s as a means of bridging the gap between laboratory science (the “bench”) and practical clinical application (the “bedside”), particularly in oncology. By the early 2000s, translational research had expanded rapidly across biomedical fields, catalyzed in part by frustration with the limited clinical yield of sustained investment in basic science. Even today, despite major investment in biomedical research, translation into therapeutic advances remains slow, with substantial attrition and reproducibility challenges. Although ophthalmic therapies have changed profoundly over the last 50 years, a substantial gap remains between experimental promise and clinical application [3].

Described below are three major research topics that originated in our research group in the spirit of basic science and subsequently acquired different translational perspectives.

### 4.1. Somatostatin—When Compelling Biology Does Not Guarantee Clinical Success

Somatostatin (Somatotropin Release-Inhibiting Factor or SRIF) appeared on our horizon simultaneously with the controversies about the Di Bella Method (DBM), proposed by the Italian physiologist Luigi Di Bella, who promoted the use of SRIF as a component of a “multitherapy” approach to cancer. In the Di Bella hypothesis, SRIF was proposed to inhibit hormonal and angiogenic signals that are potentially involved in tumor growth, but convincing clinical evidence for efficacy of the regimen was not established [10].

Investigations of the role of SRIF in the retina belong to the rapidly growing field of neuropeptide research, which is mainly focused on understanding the role of small protein-like signaling molecules acting as neuromodulators to regulate critical physiological processes. Interest in neuropeptides grew in the 1960s and 1970s with the identification of hypothalamic hormones controlling the pituitary, alongside the discovery of peptides synthesized by nerve cells and acting as neuromodulators within the nervous system. Neuropeptides were found to act as key modulators of synapses by balancing their sensitivity to excitation and inhibition through modulation of fast, primary neurotransmitters [11]. The notion that neuropeptide signaling was present in the most primitive nervous systems before the evolution of fast neurotransmitters transformed neuropeptide research. Interest further increased with evidence that neuropeptides are transiently expressed during brain development, supporting roles in synapse maturation, selection, and maintenance [12].

Neuropeptides and their receptors are expressed widely in the mammalian retina, where they exert neuromodulatory functions by dynamically adjusting the responsiveness of retinal neurons to visual stimuli [13]. Neuropeptides are mainly synthesized by amacrine and ganglion cells and act on neurons and glia, where they serve to modulate visual processing but also play a key role in developmental processes and provide neuroprotection against retinal pathologies [14].

Following major studies aimed at investigating the role of neuropeptides in retinal physiology, the possibility of translating findings from basic research into clinical applications emerged. Neuropeptides are being investigated as neuroprotective and regenerative agents for diseases such as glaucoma, DR, and ischemic injury, although randomized clinical trials are still required for many therapeutic approaches [14,15]. In the retina, neuropeptides can act as powerful endogenous neuroprotective agents by counteracting neuronal death through anti-apoptotic, anti-inflammatory, and antioxidant pathways. They may mitigate neurodegenerative damage by enhancing neurotrophin production, inhibiting oxidative stress, and reducing glutamate-induced excitotoxicity, thus offering significant therapeutic potential for retinal diseases.

Earlier studies established the retinal distribution and functional plausibility of the somatostatinergic system. SRIF is synthesized mainly by amacrine cells, while its receptors are distributed across neuronal and vascular compartments. In particular, sst2A is prominent at rod bipolar cell terminals and can modulate calcium-dependent excitability, whereas hypoxia shifts sst2A expression toward proliferating retinal endothelial cells [16,17]. These observations linked a neuromodulatory system to a plausible anti-angiogenic target.

Genetic and developmental studies further showed reciprocal regulation of sst1- and sst2-dependent signaling and effects on rod bipolar cell maturation, while sst4 signaling in retinal ganglion cells can reduce excitability and calcium loading [17,18,19,20]. These mechanistic findings were important for target validation, but the translational question became whether such biological effects could be reproduced at clinically relevant exposure and disease stages.

In the case of SRIF, the translational value of its analogs as neuroprotective factors has been supported by a series of preclinical studies showing their efficacy in protecting retinal cells from a variety of insults [21]. From the first evidence of a neuroprotective action of SRIF in DR models, in which SRIF analogs were proposed to inhibit glutamate release in the diabetic retina [21], to the more recent observation by Amato et al. [22] that SRIF analogs can protect RGCs during the early phases of DR, almost twenty years have passed without these encouraging preclinical observations resulting in an established clinical application of SRIF analogs. The implications of SRIF in DR were recently reviewed by Fang et al. [23], who discussed SRIF depletion in the very early stages of DR as a contributor to neurovascular unit impairment and microvascular damage. SRIF action at its receptors can prevent neuronal cell death through multiple interconnected cellular pathways, attenuating excitotoxicity, reducing oxidative stress, and suppressing neuroinflammation. These protective functions may be impaired when retinal SRIF content is reduced during ischemic or diabetic stress. SRIF replacement has therefore been proposed as a potential treatment for retinal neurodegeneration in DR, and work is ongoing to identify patients with early neurodegeneration who might benefit from SRIF analog supplementation [23].

The complexity of SRIF function in the retina, including its regulation of neuronal excitability and its neuroprotective action on retinal neurons, is further increased by its potent anti-angiogenic activity. GPCRs commonly exert multiple functions by triggering different cellular responses through distinct downstream pathways. Under hypoxia, dynamic changes in sst2A localization were observed: expression in retinal neuronal elements decreased, whereas sst2A became prominent in proliferating retinal endothelial cells; under normoxic conditions, retinal vessels showed little association with sst2A [24]. This hypoxia-associated endothelial localization of sst2A provides a mechanistic basis for the anti-angiogenic activity of SRIF and identifies proliferating retinal endothelial cells as a potential therapeutic target. Indeed, the neuroprotective and anti-angiogenic actions of SRIF paved the way for its potential clinical application, transforming it from a hypothalamic hormone into a potential therapeutic tool, although its rapid enzymatic degradation severely limited its clinical use. To overcome SRIF instability, Sandoz (Basel, Switzerland) initiated a program that led to the synthesis of octreotide (SMS 201-995), a stabilized cyclic octapeptide that mimics the hormone’s active region while resisting degradation. Octreotide was approved in 1988 by the US Food and Drug Administration for the treatment of acromegaly and additional neuroendocrine tumors. In preclinical models of proliferative retinopathies, octreotide was found to inhibit hypoxia-induced neovessel growth by reducing VEGF levels [25]. These findings support the concept that endogenous SRIF contributes to modulation of VEGF responses to hypoxic stress. When ischemic damage is associated with reduced retinal SRIF availability, stable SRIF analogs may therefore provide a pharmacological means of restoring part of this regulatory influence.

Signaling pathways underlying octreotide action in models of retinal vessel proliferation were described by Mei et al. [26]. Under hypoxic conditions, the signal transducer and activator of transcription 3 (STAT3) and the hypoxia-inducible factor 1 (HIF-1) cooperate to promote VEGF expression. In retinal explants, octreotide activation of sst2 prevented hypoxia-induced activation of STAT3 and HIF-1 and reduced the downstream increase in VEGF. This effect required the Src homology region 2 domain-containing phosphatase 1 (SHP-1): octreotide induced SHP-1 phosphorylation and activation, whereas SHP-1 inhibition abolished the effects of octreotide on STAT3, HIF-1, VEGF-receptor 2 (VEGFR-2), and VEGF [26]. The inhibitory effect on STAT3 was also partly indirect, through reduced VEGFR-2 phosphorylation. These findings provide a mechanistic link between sst2 activation and suppression of hypoxia-driven VEGF signaling, helping to explain the anti-angiogenic effects of octreotide in experimental retinopathy. Besides this direct retinal action, octreotide may also reduce proangiogenic drive indirectly through inhibition of growth hormone (GH) secretion and the consequent reduction in hepatic insulin-like growth factor-1 (IGF-1) production.

Although octreotide has revolutionized the management of acromegaly and neuroendocrine tumors, its therapeutic application in diabetic retinopathy has been less straightforward. Two large randomized, double-blind, placebo-controlled phase 3 programs evaluated long-acting octreotide (20 or 30 mg intramuscularly every 4 weeks) in patients with moderately severe/severe non-proliferative or low-risk proliferative diabetic retinopathy. The pooled safety population comprised 886 patients and median exposure exceeded 3.5 years; the studies were designed around the time to retinopathy progression, but the available efficacy results did not establish a consistent benefit that was sufficient for routine retinal use. Long-term treatment was generally compatible with the known octreotide safety profile, although diarrhea, cholelithiasis and hypoglycemia were among the more frequent adverse events [27]. The later EUROCONDOR randomized trial enrolled 449 patients with type 2 diabetes and no or early diabetic retinopathy and compared somatostatin 0.1% (one drop twice daily in each eye), brimonidine, and placebo for 96 weeks. Somatostatin did not improve the primary multifocal electroretinography endpoint in the overall population, although worsening appeared to be prevented in the subgroup with pre-existing retinal neurodysfunction [23,28]. Thus, the trial generated a hypothesis about patient selection rather than evidence for general clinical efficacy. The endocrine rationale also remains biologically plausible because GH/IGF-1 signaling has been associated with retinal neovascular processes [29], but biological plausibility alone did not overcome the limitations encountered in clinical translation. A more recent study of prolonged-release lanreotide (90 mg subcutaneously every 4 weeks for 12 months) in refractory DME reported an improvement in visual acuity, but it was a small, non-randomized study and therefore cannot establish efficacy comparable with controlled anti-VEGF trials [30]. Collectively, these findings suggest that dose, route, disease stage, patient selection and endpoint choice may all have contributed to the gap between the strong biological rationale and the limited clinical translation of somatostatin analogs.

### 4.2. uPA/uPAR and UPARANT—When a Drug Does Not Reach the Clinic but Its Biology Remains Valuable

If VEGF orchestrates the proliferation of retinal endothelial cells, their subsequent tube formation requires coordinated cell migration, extracellular matrix (ECM) degradation, and specific cytoskeletal rearrangements. In this respect, the system formed by the ligand urokinase-type plasminogen activator (uPA) and its receptor uPAR drives several biological mechanisms, including ECM degradation, cell adhesion and migration, and intracellular signaling independent of the protease activity of uPA. In the retina, uPAR is primarily localized to endothelial cells lining the blood vessels in the superficial layers and to the basolateral membrane of the retinal pigment epithelium [31]. Under hypoxic conditions, including those occurring in ischemic retinopathies, uPAR is highly expressed in endothelial cells, particularly in proliferating capillaries and neovascular tufts. Work investigating the anti-angiogenic and anti-inflammatory potential of the peptide UPARANT helped to clarify mechanisms associated with the uPA/uPAR system. As reviewed by Cammalleri et al. [31], UPARANT can counteract pathological retinal angiogenesis by interfering with formyl peptide receptor- (FPR-) and integrin-dependent signaling linked to uPAR activity, rather than by directly inhibiting uPA binding to uPAR. Although UPARANT did not reach the clinical stage, elucidation of the mechanisms mediating its inhibitory action on angiogenesis and inflammation helped us to clarify the role of the uPAR system as a key player at the intersection between inflammatory and angiogenic responses to ischemic injury. Whereas octreotide acts upstream by limiting hypoxia-induced VEGF expression through sst2 signaling, UPARANT acts downstream of VEGF upregulation by interfering with signaling pathways that control endothelial cell proliferation and migration, fundamental processes driving new blood vessel formation and vascular repair. Once upregulated, VEGF binds with high affinity to VEGFR-2 on the surface of endothelial cells and promotes proliferation and migration. Endothelial invasion also requires activation of proteolytic enzymes that degrade the basement membrane and ECM, allowing migration through the remodeled matrix. As detailed by Cammalleri et al. [31], the protease system formed by uPA and uPAR plays a central role in angiogenesis. uPA is a serine protease produced and secreted by many cell types, including endothelial cells. uPA binding to uPAR increases the conversion of plasminogen into plasmin, which contributes to ECM breakdown either directly or indirectly by activating matrix metalloproteinases (MMPs). Extracellularly, uPAR interaction with vitronectin initiates signaling cascades that regulate integrin signaling and VEGF/VEGFR-2 coupling. Functional interaction between uPAR and FPRs, lateral partners for signal transduction belonging to the GPCR family, activates multiple signaling pathways that mediate cellular responses including endothelial cell proliferation. In particular, uPAR/FPR signaling can activate the phosphatidylinositol 3-kinase (PI3K)/protein kinase B (AKT) pathway and promote endothelial cell proliferation.

In vivo models of retinal vessel proliferation showed that UPARANT inhibited neovascular responses to hypoxia by interfering with FPR- and integrin-associated signaling in the uPAR network [31,32]. UPARANT also prevented neovessel proliferation induced by vitreous fluid from patients with PDR, supporting activity against a complex mixture of proangiogenic and inflammatory stimuli [32]. In the rd10 model of retinitis pigmentosa, it reduced degenerative changes and improved retinal function through signaling partners associated with the uPAR network [33]. Importantly, UPARANT progressed beyond efficacy studies to preclinical toxicological evaluation, but development was subsequently discontinued before clinical testing. According to the author’s direct knowledge of the development program, this decision was strategic and commercial: the company responsible for the compound judged the regulatory pathway to be complex and costly and elected not to continue the registration process. Thus, the absence of clinical development should not be interpreted as evidence that the biological target or preclinical efficacy had failed. At the same time, because no human trial was performed, clinical efficacy, optimal exposure and human safety remain unknown. The pathway nevertheless remained scientifically productive: N-19004, a newer FPR antagonist related functionally and structurally to UPARANT [34], preserved photoreceptor resilience and retinal function in rd10 mice [35], and inhibition of uPAR/FPR1 interactions reduced blood–retinal barrier breakdown and improved retinal function in experimental diabetes [36].

### 4.3. β-Adrenoceptors and Propranolol—When Repurposing Shortens the Translational Path

With respect to drug discovery, drug repurposing is a fast and cost-effective strategy for identifying new therapeutic uses for existing approved or investigational compounds. By leveraging established safety data, this approach can reduce the development time and risk compared with traditional drug discovery. Repositioning a drug has several advantages: shorter development time, lower costs, the availability of existing human safety and toxicity data, potential application to rare or orphan diseases that might not justify the investment required for traditional drug development, and the possibility of identifying new uses among the large number of compounds with known pharmacological activity. There are several examples of drug repositioning. In the eye, a prime example is bevacizumab (Avastin), which was originally developed for cancer and was subsequently found to be highly effective for treating AMD [5]. In addition, population studies and advanced bioinformatics have identified possible opportunities for AMD repurposing among agents that have already been approved for other indications, including metformin, high-dose statins, levodopa (L-DOPA), and fluoxetine [37].

In retinal neovascular disease, drug repurposing offers interesting perspectives, including propranolol, an antagonist of β1/β2 adrenoceptors (β1/2-ARs) already used in infantile hemangioma (IH). Propranolol is the first-line pharmacological treatment for infantile hemangioma, the most common benign vascular tumor of infancy, whose growth involves angiogenic and hypoxia-responsive pathways. Propranolol acts as a non-selective blocker of β1/2-ARs. Hypoxia-driven sympathetic activation can increase norepinephrine (NE) signaling, which in turn may promote proangiogenic pathways. As a major pathogenetic mechanism of IH, pathological angiogenesis involves degradation of the basement membrane followed by proliferation, migration, and aggregation of activated hemangioma-derived endothelial cells to form neovasculature. On the one hand, hypoxia promotes the release of growth factors, including VEGF, that stimulate blood vessel growth. On the other hand, hypoxia-driven adrenergic signaling can contribute to VEGF overexpression through HIF-1-dependent pathways. The striking similarities between neovessel proliferation in IH and the proliferative phase of ROP prompted us to launch a series of investigations that culminated in clinical trials exploring propranolol as a new therapeutic strategy in ROP. Mechanistically, sympathetic overstimulation in response to hypoxia may lead to increased release of NE in the choroidal circulation, from which it can influence β-ARs in the retina. Among these receptors, β2-AR is a relevant propranolol target and is expressed by Müller cells, important retinal sources of VEGF. In the OIR model mimicking ROP, systemic propranolol was found to inhibit retinal vessel proliferation and blood–retinal barrier breakdown by reducing proangiogenic factors [38]. β2-AR signaling can activate cAMP/PKA and other downstream pathways that converge on HIF-1α and VEGF expression [38,39]. β2-AR blockade by propranolol interrupts this proangiogenic signaling, reducing HIF-1α accumulation and downstream VEGF expression and thereby limiting pathological neovascularization. However, the preclinical evidence was not completely uniform. Chen et al. reported that propranolol did not suppress pathological neovascularization in OIR. In a contemporaneous IOVS letter, we noted that a major difference between their study and ours was the mouse strain: Chen et al. used the 129S6/SvEvTac background, whereas our propranolol studies used C57BL/6J mice, and we proposed that strain-dependent angiogenic and adrenergic responses could contribute to the discrepant findings [40]. This observation later acquired additional mechanistic relevance. Using the related 129S genetic background, Melecchi et al. found a more pronounced hypoxia-driven angiogenic response together with marked β3-AR upregulation; in this setting, β3-AR agonism promoted physiological revascularization and limited pathological neovascularization, whereas previous studies in C57BL/6J mice had not revealed a significant effect of β3-AR pharmacology [41]. These findings do not prove that β3-AR expression alone explains the discrepancy in the propranolol studies, but they reinforce the broader point that genetic background and receptor expression can strongly influence pharmacological responses in OIR.

Since 2011, clinical studies have examined whether β-adrenoceptor blockade can limit ROP progression. In a randomized study of 52 preterm infants with stage 2 ROP, oral propranolol (0.25 or 0.5 mg/kg every 6 h) was associated with less progression to severe ROP, but systemic administration raised clinically important safety concerns in this fragile population [42]. A subsequent multicenter phase II study of 0.1% propranolol eye micro-drops in 23 infants with stage 2 ROP showed good tolerability but insufficient efficacy [43]. Increasing the topical concentration to 0.2% and treating earlier-stage disease was then evaluated in a multicenter open-label phase IIB study: 98 newborns with stage 1 ROP were compared with historical controls, and progression to stage 2 or 3 plus disease was lower than expected (risk ratio 0.521, 95% CI 0.297–0.916), with no adverse effects attributed to propranolol [44]. However, the open-label design and use of historical controls limit the strength of the efficacy inference. These studies therefore support continued investigation rather than routine clinical use.

The propranolol experience also illustrates the need to separate what an experimental model reproduces well from what it does not. The mouse oxygen-induced retinopathy (OIR) model reproduces a biphasic sequence of hyperoxia-associated vaso-obliteration followed, after return to room air, by relative retinal hypoxia, HIF-1/VEGF activation and pathological neovascularization. It is therefore particularly useful for dissecting hypoxia-driven angiogenic signaling and testing interventions during the proliferative phase. In mouse OIR, vaso-obliteration is predominantly central, whereas human ROP is characterized by a peripheral avascular retina. Moreover, mice lack a macula, and OIR models differ in the vascular compartments affected by hypoxia, since OIR mice involve blood vessels sprouting from the retinal rather than choroidal neovascularization, which further limits direct extrapolation to nAMD. However, it does not reproduce the full systemic context of prematurity, the variable oxygen exposures and comorbidities of human ROP, the chronic metabolic and inflammatory injury of diabetic retinopathy, or the aging, retinal pigment epithelium, choroidal and complement-related biology of nAMD. Shared activation of VEGF or related pathways therefore supports mechanism-oriented comparisons but does not imply that the same intervention, dose, timing or route will translate across these diseases [4,45].

Within the β-AR system, the clinical use of the β3-AR agonist mirabegron for an overactive bladder (OAB) drew our attention to the possible retinal effects of β3-AR activation [46]. In the OIR model, β3-AR agonism has been shown to influence neovessel proliferation in response to ischemic injury by promoting revascularization of the central avascular retina after hyperoxia. These studies exploited the 129S background, in which hypoxia induces a particularly strong angiogenic response together with marked β3-AR upregulation, making this strain especially informative for testing β3-AR agonism [41]. This reduces the hypoxic stimulus in the midperiphery and consequently limits pathological neovessel overgrowth [41,46]. In this respect, a study protocol examining AMD progression in patients receiving mirabegron for overactive bladder (OAB) has recently been published [47]. Further support for the therapeutic potential of β3-AR agonism comes from findings showing that a reduction in the central avascular area occurs together with restoration of the astrocytic template, underlining the contribution of astrocytes to retinal protection and opening a new cycle of hypothesis generation and repurposing [41]. In Table 1, the three translational trajectories were compared, while in Table 2, their quantitative and design features were shown.

## 5. Drug Delivery and Exposure: The Often-Underestimated Translational Filter

Target validation is necessary to establish a biological link between a drug candidate and its functional efficacy. However, a drug must reach the relevant retinal compartment at an effective concentration, for an adequate period of time, without unacceptable systemic or local toxicity. This is why anti-VEGF treatments are administered intravitreally, despite the limitations of this route. Pharmacological targeting of the posterior segment requires bypassing physiological barriers that protect internal tissues from pathogens, foreign bodies and toxins [48]. Topical delivery is attractive because it is non-invasive, but rapid drainage and limited posterior-segment penetration can reduce exposure; systemic delivery may reach the retina but can require higher circulating concentrations and increase off-target effects. In our mouse OIR studies, repeated systemic administration was often the only practical option because repeated intravitreal injections are difficult in the very small developing eye. Such practical success in an experimental model should not be confused with a demonstration of an optimal human route. For octreotide, systemic administration produced reproducible sst2-dependent anti-angiogenic effects in OIR [24,25], yet clinical studies in diabetic retinopathy were inconsistent [27], illustrating how exposure, disease stage and patient selection can alter translation.

Downstream of the hypoxia-associated upregulation of the VEGF system, we devoted substantial effort to exploring the efficacy of subcutaneous administration of compounds acting on signaling cascades that control endothelial cell proliferation and migration. At the preclinical level, UPARANT, which interferes with FPR- and integrin-associated signaling in the uPAR network [31], was shown to reduce processes underlying pathological retinal vessel proliferation, indicating activity at the retinal level after systemic administration [32]. As reported by Cammalleri et al. [31], in a model of DR, UPARANT remained detectable in plasma up to 24 h after subcutaneous injection, consistent with its high plasma stability and prolonged systemic exposure. Two hours after administration, UPARANT was detectable in the eye and remained detectable at 24 h, when its level was approximately 20% of that measured at 2 h. In the retina, the UPARANT level was about 15% of that measured in the whole eye, suggesting that the drug can cross the blood–retinal barrier. Retinal entry may be facilitated in DR by blood–retinal barrier breakdown and the associated increase in vascular permeability.

In the context of systemic delivery, additional preclinical studies in the OIR model demonstrated that propranolol, a non-selective β1/2-AR blocker, was effective in preventing retinal neovascularization and blood–retinal barrier breakdown after subcutaneous administration. However, repeated subcutaneous administration of propranolol is impractical in preterm infants with ROP. Therefore, pilot randomized trials evaluated the safety and efficacy of oral propranolol in preterm newborns with an early phase of ROP [42]. Although safety concerns emerged in the pilot studies [42], subsequent meta-analyses supported an effect of oral propranolol on ROP progression while confirming the need for careful safety assessment [49]. Concurrently, investigation of propranolol pharmacokinetics in pediatric patients required methods based on less invasive sampling and minimal blood volumes. Therefore, a micromethod for measuring propranolol levels on dried blood spots was developed using liquid chromatography/tandem mass spectrometry [50,51]. To reduce the systemic toxicity associated with oral propranolol, topical administration was explored first in preclinical models and then clinically. In the OIR model, topical propranolol counteracted pathological neovascularization [52]. Although direct measurement of retinal exposure remained challenging, modulation of PKA activity in retinal homogenates indicated an effect on the retinal cAMP/PKA axis [52]. In terms of efficacy, topical administration at 2% produced effects comparable to those of 20 mg/kg propranolol administered either subcutaneously or per os, with a plasma distribution similar to that obtained after oral administration [52]. In clinical trials, 0.1% propranolol eye micro-drops administered to preterm infants were well tolerated but not sufficiently effective [43], whereas 0.2% eye micro-drops were well tolerated and were associated with reduced ROP progression compared with historical controls [44,53]. Because the 0.2% phase IIB study was open-label and used a historical control group, these findings require confirmation in randomized placebo-controlled trials [44]. Although propranolol has not yet been established as routine therapy for ROP, the available clinical and preclinical evidence supports continued evaluation of β1/2-AR blockade as a strategy to limit hypoxia-driven pathological angiogenesis [45,54].

## 6. Why Promising Preclinical Findings Fail to Reach the Bedside

The three trajectories considered here illustrate a broader translational problem. Promising preclinical findings can fail because the experimental model captures only part of human disease, because reproducibility is limited, because the intervention is tested at the wrong disease stage, or because pharmacokinetics, tissue exposure, toxicity, patient heterogeneity and endpoint selection differ between experimental and clinical settings. Developmental feasibility and commercial considerations can also interrupt a program independently of biological plausibility, as occurred with UPARANT. Human-derived systems, organoids and in silico approaches can strengthen target validation, but none fully reproduces whole-patient complexity [3,55]. Translational decisions should therefore integrate mechanistic coherence, independent replication, exposure-response information, clinically meaningful endpoints and a realistic development pathway, rather than relying on a single positive efficacy signal.

## 7. Basic Science and Translational Research: Not Competing Priorities

Basic and translational research should therefore be viewed as complementary stages of the same scientific cycle. Curiosity-driven studies identify mechanisms and unexpected targets; translational studies then test whether those mechanisms remain actionable under constraints of dose, delivery, safety and disease heterogeneity. Negative clinical or developmental outcomes can in turn expose weaknesses in the original model and generate new basic questions. The somatostatin, uPA/uPAR and β-adrenoceptor programs each followed this cycle in different ways: one reached controlled clinical testing without becoming routine care, one stopped before human trials for non-biological developmental reasons, and one advanced through repurposing into early clinical investigation.

## 8. What Forty Years of Retinal Research Taught Me About Translation

Although these lessons derive from retinal research, they are not retina-specific. Across translational biomedicine, model validity should be defined in relation to the mechanism being tested rather than assumed from superficial disease similarity; reproducibility should precede the escalation of development; pharmacokinetic exposure must be demonstrated at the relevant tissue; disease stage and patient selection should match the mechanism of action; safety must be evaluated in the population actually intended for treatment; and endpoints must reflect a clinically meaningful benefit. These principles help explain why strong target biology can coexist with variable clinical outcomes and why failure at one translational step does not necessarily invalidate the underlying mechanism. They also argue for bidirectional translation, in which clinical observations and unsuccessful development programs are used to refine experimental questions.

## 9. Conclusions—From Bench to Bedside, and Sometimes Back Again

**Figure 2:**
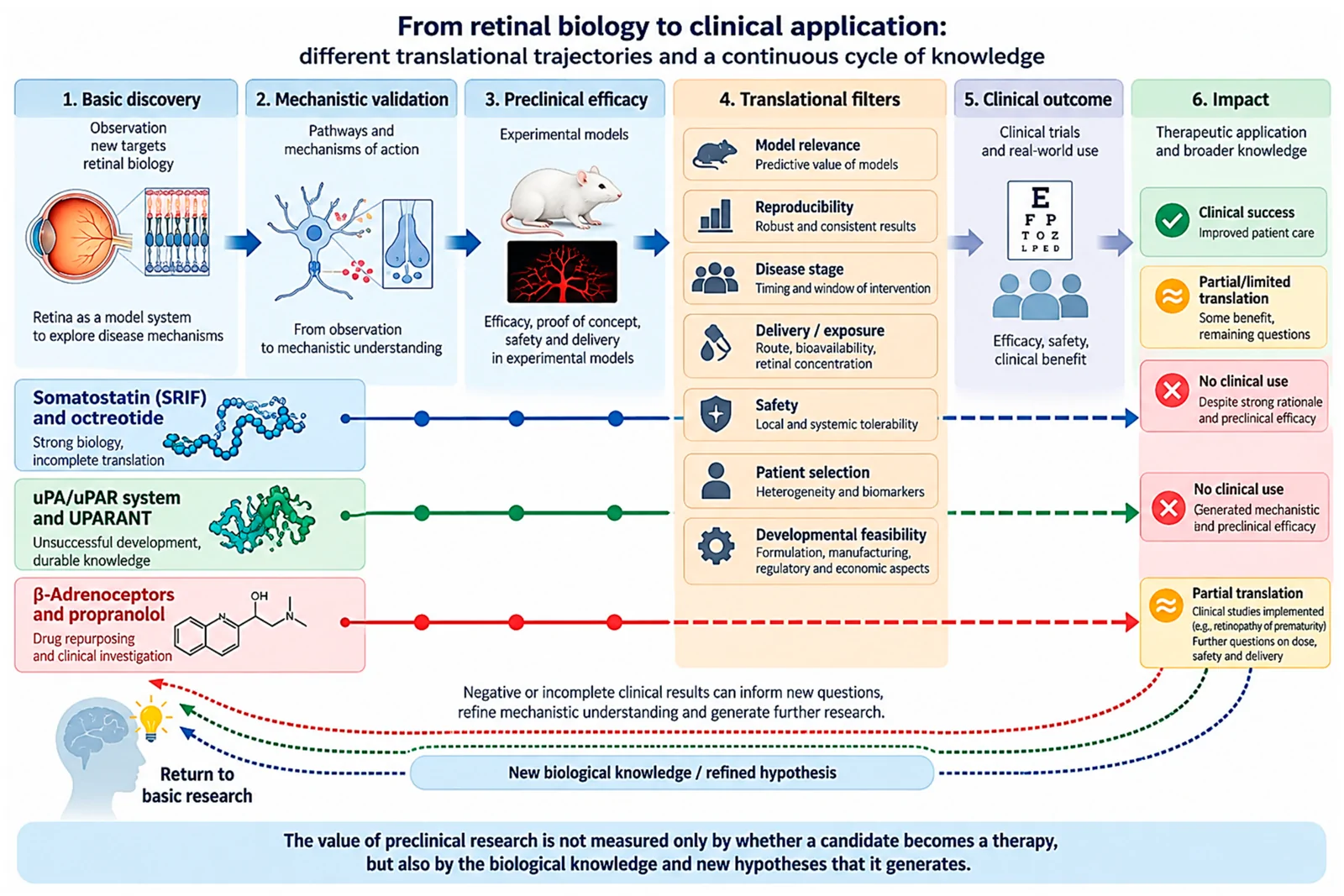
Translational pathway from retinal basic research to clinical application. The three research programs illustrate different translational outcomes, ranging from controlled clinical testing without routine adoption to preclinical development discontinued before human trials and to early clinical investigation through drug repurposing. Negative, incomplete or strategically discontinued programs may still generate new biological knowledge and refined hypotheses, feeding back into basic research and emphasizing the iterative rather than linear nature of translation.

Whether a preclinical candidate ultimately becomes a type of therapy is an important measure of success, but it is not the only one. The examples discussed here show that translation is a sequence of filters rather than a one-way road: mechanistic validity, model relevance, reproducibility, exposure, safety, disease stage, patient selection, clinically meaningful endpoints and developmental feasibility all influence whether a promising observation can become useful treatment. When translation is incomplete, the resulting information can still refine biological understanding and redirect subsequent work. Preserving basic curiosity-driven research is therefore essential, while translational programs should be judged with equal attention to scientific rigor and practical feasibility. Figure 2 summarizes this iterative relationship between discovery, preclinical validation, translational filters and answers to new biological questions.

## Figures and Tables

**Table 1:** Comparison of the three translational trajectories discussed in the manuscript.

Main Lesson	Translational Outcome	Preclinical Rationale/Evidence	Research Program
Strong biology does not guarantee clinical efficacy; stage, route, exposure, patient selection and endpoints matter.	Reached randomized clinical testing; phase 3 octreotide and EUROCONDOR did not establish routine retinal use. Small later studies remain exploratory.	Retinal neuromodulation, neuroprotection, and sst2-dependent inhibition of hypoxia/VEGF signaling.	Somatostatin/octreotide
A biologically productive program can stop for non-scientific developmental reasons; absence of clinical testing is not evidence of target failure, but human efficacy and safety remain unknown.	Reached preclinical toxicology but not human trials; development was discontinued for strategic/commercial reasons rather than because of demonstrated loss of biological efficacy.	Interference with FPR- and integrin-associated signaling in the uPAR network reduced angiogenesis, inflammation and retinal degeneration in experimental models.	uPA/uPAR/UPARANT
Repurposing can shorten development but does not remove disease-specific pharmacological and clinical uncertainty.	Progressed from OIR studies to oral and topical clinical investigation in ROP; oral dosing raised safety concerns, 0.1% micro-drops were insufficiently effective, and 0.2% data remain non-randomized.	Adrenergic control of hypoxia-driven angiogenesis in OIR suggested repurposing of an established β-blocker; however, the antiangiogenic effect was not reproduced in every OIR study, with mouse-strain differences providing a plausible contributor to the discrepancy.	β-adrenoceptors/propranolol

**Table 2:** Representative quantitative and design features of the principal translational studies discussed in the manuscript.

Intervention/Context	Design and Population	Dose/Route	Representative Endpoint/Outcome	Key Limitation/Safety/Status
Long-acting octreotide in DR [27]	Two randomized, double-blind, placebo-controlled phase 3 programs; pooled safety n = 886; moderate/severe NPDR or low-risk PDR.	20 or 30 mg intramuscularly every 4 weeks.	Designed around time to retinopathy progression; available results did not establish consistent efficacy sufficient for routine use.	Median exposure >3.5 years; diarrhea, cholelithiasis and hypoglycemia among common adverse events; no routine retinal indication.
Topical somatostatin (EUROCONDOR) [28]	Randomized multicenter trial; 449 patients with type 2 diabetes and no/early DR; somatostatin n = 145, placebo n = 152.	Somatostatin 0.1%, one drop twice daily in each eye, for 96 weeks.	Primary mfERG implicit-time endpoint negative in the overall population; subgroup with baseline neurodysfunction showed less worsening.	Post hoc/subgroup signal; supports patient stratification hypothesis rather than established efficacy.
Lanreotide in refractory DME [30]	Small non-randomized clinical study; 32 eyes of 18 patients with DME resistant to conventional therapy.	90 mg prolonged-release subcutaneous injection every 4 weeks for 12 months.	Mean visual-acuity improvement reported (about 8.6 ETDRS letters).	No randomized control; selected refractory population; insufficient for routine-care conclusions.
UPARANT [31,32,33]	Preclinical retinal efficacy studies followed by toxicological evaluation; no human trial.	Systemic/subcutaneous administration in experimental models.	Reduced pathological angiogenesis/inflammation in OIR and supported retinal protection in other disease models.	Development stopped before clinical testing for strategic/commercial reasons; human dose, efficacy and safety remain unknown.
Oral propranolol in ROP [42]	Randomized study; 52 preterm infants with stage 2 ROP.	0.25 or 0.5 mg/kg orally every 6 h.	Lower progression to severe ROP was reported.	Systemic safety concerns in preterm infants limited this route.
Propranolol 0.1% micro-drops [43]	Multicenter open-label phase II pilot; 23 infants with stage 2 ROP.	Topical 0.1% eye micro-drops.	Progression remained about 26%; predefined efficacy target not met.	Well tolerated but insufficiently effective.
Propranolol 0.2% micro-drops [44]	Multicenter open-label phase IIB; 98 infants with stage 1 ROP compared with historical controls.	Topical 0.2% eye micro-drops.	Progression to stage 2 or 3 plus lower than expected; RR 0.521 (95% CI 0.297–0.916).	No treatment-related adverse effects reported, but historical controls and open-label design limit inference.

## Data Availability

No new data were created or analyzed in this study. Data sharing is not applicable to this article.

## Abbreviations

The following abbreviations are used in this manuscript:

age-related macular degeneration	**AMD**	protein kinase B	**AKT**
β-adrenoceptor(s) and indicated receptor subtypes	**β-AR(s); β1/β2-ARs; β2-AR; β3-AR**	angiopoietin-2	**Ang-2**
choroidal neovascularization	**CNV**	cyclic adenosine monophosphate	**cAMP**
diabetic macular edema	**DME**	Di Bella Method	**DBM**
extracellular matrix	**ECM**	diabetic retinopathy	**DR**
formyl peptide receptor; formyl peptide receptor 1	**FPR; FPR1**	European Consortium for the Early Treatment of Diabetic Retinopathy	**EUROCONDOR**
ganglion cell layer	**GCL**	gamma-aminobutyric acid	**GABA**
G-protein-coupled receptor	**GPCR**	growth hormone	**GH**
Ca2+-activated K+ current	**IBK**	hypoxia-inducible factor 1	**HIF-1**
infantile hemangioma	**IH**	insulin-like growth factor 1	**IGF-1**
inner plexiform layer	**IPL**	inner nuclear layer	**INL**
matrix metalloproteinases	**MMPs**	levodopa (L-3,4-dihydroxyphenylalanine)	**L-DOPA**
norepinephrine	**NE**	neovascular age-related macular degeneration	**nAMD**
oxygen-induced retinopathy	**OIR**	overactive bladder	**OAB**
proliferative diabetic retinopathy	**PDR**	optic nerve	**ON**
protein kinase A	**PKA**	phosphatidylinositol 3-kinase	**PI3K**
rod bipolar cell	**RBC**	Ras-related C3 botulinum toxin substrate 1	**Rac1**
retinopathy of prematurity	**ROP**	retinal ganglion cell	**RGC**
Src homology region 2 domain-containing phosphatase 1	**SHP-1**	retinitis pigmentosa	**RP**
somatostatin receptor(s) and indicated receptor subtypes	**sst(s); sst1; sst2; sst2A; sst3; sst4; sst5**	somatotropin release-inhibiting factor	**SRIF**
urokinase-type plasminogen activator	**uPA**	signal transducer and activator of transcription 3	**STAT3**
uPAR-derived peptide inhibitor (cenupatide)	**UPARANT**	urokinase-type plasminogen activator receptor	**uPAR**
vascular endothelial growth factor receptor 2	**VEGFR-2**	vascular endothelial growth factor and indicated isoforms	**VEGF; VEGF-A; VEGF-B**
systematic evolution of ligands by exponential enrichment	**SELEX**	polyethylene glycol	**PEG**
